# Development and Application of Biosensors in the Food Field

**DOI:** 10.3390/foods15081430

**Published:** 2026-04-20

**Authors:** Rui Chen, Sihang Zhang, Hongji Li, Long Wu

**Affiliations:** 1 Analysis and Testing Center, Chinese Academy of Tropical Agricultural Sciences, Haikou 571101, China; chenrui9505@126.com; 2 School of Food Science and Engineering, Hainan University, Haikou 570228, China; sih.zhang@hainanu.edc.cn; 3 College of Chemistry and Chemical Engineering, Hainan Normal University, Haikou 571158, China; hongjili1102@hainnu.edu.cn

The assurance of food safety is essential for public health and environmental sustainability. However, rapid industrialization and intensive agricultural practices have increased the prevalence of chemical contaminants within the food supply chain. Hazards associated with food spoilage, such as mycotoxins, pesticide residues, heavy metals, and biogenic amines, present significant risks to consumers and ecosystems. Although conventional analytical techniques such as high-performance liquid chromatography (HPLC) and gas chromatography–mass spectrometry (GC-MS) offer excellent sensitivity and precision, their practical application remains limited. They depend on costly instrumentation, lengthy sample pretreatment, and specialized operators, which restricts their use in rapid, on-site, and non-destructive analyses [1]. Consequently, developing efficient, portable, and intelligent sensing platforms has emerged as a critical objective in analytical chemistry and food science.

The continuous development of nanotechnology and functional materials has facilitated the design of high-performance sensing platforms [2]. Because of their unique physicochemical properties, a growing number of nanomaterials, including graphitic carbon nitride, carbon dots, metal nanoclusters, and upconversion nanoparticles, are being used for signal amplification and bioreceptor immobilization. These applications are driven by their high specific surface area, tunable surface chemistry, and robust biocompatibility [3,4]. Furthermore, integrating advanced recognition strategies, including molecular imprinting, nucleic acid aptamers, and enzyme-mimetic catalysis, has substantially improved sensor selectivity and stability [5,6]. To address analytical difficulties such as chiral recognition, trace analyte enrichment in complex matrices, and multiplexed detection, current research focuses on synergistic composite materials and stimuli-responsive smart probes.

Concurrently, the integration of artificial intelligence and mobile communication technologies has transformed sensor systems. Smartphone-based image processing and deep learning algorithms now enable the automated acquisition and analysis of optical signals, such as colorimetric and fluorometric changes [7,8]. This integration significantly facilitates the miniaturization and portability of analytical devices. Furthermore, exploiting photophysical mechanisms, including fluorescence polarization, the inner filter effect, and aggregation-induced emission, has guided the development of rapid, separation-free homogeneous sensing platforms. The convergence of these innovations systematically overcomes the operational constraints of conventional methods. By reducing costs without sacrificing sensitivity, these integrated platforms bridge the gap between laboratory analysis and on-site screening, accommodating both professional analysts and general consumers.

This Special Issue focuses on the development and application of biosensors in the food field, bringing together a collection of innovative research findings that cover multiple cutting-edge directions, ranging from the design of novel nanoprobes to the construction of intelligent detection platforms. For example, a molecularly imprinted surface plasmon resonance (SPR) sensor based on sulfur-doped boron graphitic carbon nitride (S-B-g-C_3_N_4_) achieved ultra-trace detection of the mycotoxin deoxynivalenol (DON) in beverages, with detection limits as low as 0.30 ng L^−1^ (Contribution 1). Another study sustainably transformed coffee ground waste into fluorescent iron-doped carbon dots (Fe-CDs), enabling a dual-channel colorimetric/fluorescent sensor array for assessing total antioxidant capacity in foods (Contribution 2). Similarly, glutathione-stabilized copper nanoclusters (GSH-Cu NCs) were developed as a simple “switch-off” fluorescent sensor for quercetin detection in tea samples, achieving a detection limit of 24 Nm (Contribution 3).

Chirality and selectivity, which are critical for ensuring food safety and nutritional authenticity, can be addressed by using a novel chiral molecularly imprinted electrochemical sensor incorporating β-cyclodextrin-functionalized graphene quantum dots (GQDs/β-CD) (Contribution 4). This sensor demonstrates enantioselective recognition of D-carnitine with an extraordinary detection limit of 2.35 × 10^−13^ mol/L and a 7.15-fold enhancement in selectivity. An aptamer-based fluorescence biosensor using alendronic acid-modified upconversion nanoparticles (UCNPs) combined with magnetic separation enables the rapid (25 min) and sensitive detection of the pesticide thiamethoxam in cucumber, cabbage, and apple samples, with a detection limit of 0.08 ng·mL^−1^ (Contribution 5).

Beyond traditional target analytes, this Special Issue also highlights innovative approaches to assessing food quality attributes and enabling consumer-level testing. For the first time, a fluorescence polarization (FP) assay using a chitooligosaccharide tracer was established to determine lysozyme activity in egg whites from multiple bird species, facilitating the selection of eggs with high active lysozyme content for long-term storage (Contribution 6). Perhaps most impressively, a smartphone-based multimodal deep learning approach—termed FreshFusionNet—was developed using pH-sensitive pitaya peel films for the real-time non-destructive detection of fish freshness (Contribution 7). By integrating image data from multiple angles and lighting conditions with chemical indicators (pH, TVB-N, TVC) via temporal convolutional networks and a context-aware gated fusion mechanism, the system achieved 99.61% classification accuracy on a commercial smartphone, demonstrating the significant potential of intelligent, portable devices for democratizing food safety monitoring.

Finally, a comprehensive review included in this Special Issue critically evaluated the state-of-the-art detection technologies for pesticide residues and heavy metals in rice, covering spectroscopy, chromatography, immunoassays, and biosensors. This review underscores the pressing need for miniaturization, multiplexed detection, nanotechnology integration, and real-time monitoring systems, providing a theoretical roadmap for future innovations in the field (Contribution 8).

Collectively, the contributions to this Special Issue illustrate that biosensors are rapidly maturing from proof-of-concept studies into practical, sustainable, and intelligent tools. They address the key challenges highlighted in our initial solicitation, including sensor stability, reproducibility, cost-effectiveness, and integration with digital platforms. Additionally, they open new avenues for on-site, real-time, and consumer-accessible food analysis. We extend our sincere gratitude to all of the authors for their outstanding contributions, to the reviewers for their rigorous and constructive evaluations, and to the editorial team of *Foods* for their unwavering support. We trust that readers will find this Special Issue both inspiring and practically useful, and we hope that it will catalyze further interdisciplinary collaborations that continue to revolutionize food safety and quality assurance in the years to come.

## References

[1] Guo N., Yang J., Li Y., Wang W., Liang X., Xu Q., Du L., Qin J. (2025). A review of a colorimetric biosensor based on Fe_3_O_4_ nanozymes for food safety detection. Anal. Bioanal. Chem..

[2] Darwish M.A., Abd-Elaziem W., Elsheikh A., Zayed A.A. (2024). Advancements in nanomaterials for nanosensors: A comprehensive review. Nanoscale Adv..

[3] Zargul A., Liu H., Zhang W., Wang H., Liu J., Chen C. (2026). Advances in Pathogen Detection by Nanosensors: Bio-recognition Strategies, Signal Amplification, and Platform Engineering. ACS Nano.

[4] Kim J.Y., Kim M.Y., Song Y., Oh M.J., Bong J.H., Park M. (2026). Recent Strategies in Nanomaterials-Based Signal Amplification of Electrochemical Biosensors. BioChip J..

[5] Hu G., Zhao M., Zhuang Y., Yu P., Zhang D., Gao S., Hao J. (2025). Research on the application of molecular imprinting technology in the detection of mycotoxins and aquatic biotoxins. Microchem. J..

[6] Zhang L., He X., Hu J., Bai H., Yao Y., Hu W.W. (2025). Recent advances in nanozymes-CRISPR/Cas biosensors. Chem. Commun..

[7] Nayak A., Chakraborty S., Swain D.K. (2023). Application of smartphone-image processing and transfer learning for rice disease and nutrient deficiency detection. Smart Agric. Technol..

[8] Principi N., Esposito S. (2024). Smartphone-Based Artificial Intelligence for the Detection and Diagnosis of Pediatric Diseases: A Comprehensive Review. Bioengineering.

